# Impact of *KRAS, BRAF, PIK3CA* Mutations, PTEN, *AREG, EREG* Expression and Skin Rash in ≥2^nd^ Line Cetuximab-Based Therapy of Colorectal Cancer Patients

**DOI:** 10.1371/journal.pone.0015980

**Published:** 2011-01-20

**Authors:** Zacharenia Saridaki, Maria Tzardi, Chara Papadaki, Maria Sfakianaki, Fraga Pega, Aristea Kalikaki, Eleftheria Tsakalaki, Maria Trypaki, Ippokratis Messaritakis, Efstathios Stathopoulos, Dimitris Mavroudis, Vassilis Georgoulias, John Souglakos

**Affiliations:** 1 Laboratory of Tumor Cell Biology, School of Medicine, University of Crete, Heraklion, Crete, Greece; 2 Department of Medical Oncology, University General Hospital of Heraklion, Heraklion, Crete, Greece; 3 Laboratory of Pathology, University General Hospital, Heraklion, Crete, Greece; Wistar Institute Program, United States

## Abstract

**Background:**

To investigate the predictive significance of *KRAS*, *BRAF, PIK3CA* mutational status, *AREG- EREG* mRNA expression, PTEN protein expression and skin rash in metastatic colorectal cancer (mCRC) patients treated with cetuximab containing salvage chemotherapy.

**Methods:**

Primary tumors from 112 mCRC patients were analyzed. The worst skin toxicity during treatment was recorded.

**Results:**

*KRAS*, *BRAF* and *PIK3CA* mutations were present in 37 (33%), 8 (7.2%) and 11 (9.8%) cases, respectively, PTEN was lost in 21 (19.8%) cases, *AREG* and *EREG* were overexpressed in 48 (45%) and 51 (49%) cases. In the whole study population, time to tumor progression (TTP) and overall survival (OS) was significantly lower in patients with *KRAS* (*p* = 0.001 and *p* = 0.026, respectively) or *BRAF* (*p* = 0.001 and *p*<0.0001, respectively) mutant tumors, downregulation of *AREG* (*p* = 0.018 and *p* = 0.013, respectively) or *EREG* (*p* = 0.002 and *p* = 0.004, respectively) and grade 0-1 skin rash (*p*<0.0001 and *p*<0.0001, respectively). In *KRAS* wt patients TTP and OS was significantly lower in patients with *BRAF* (*p* = 0.0001 and *p*<0.0001, respectively) mutant tumors, downregulation of *AREG* (*p* = 0.021 and *p* = 0.004, respectively) or *EREG* (*p* = 0.0001 and *p*<0.0001, respectively) and grade 0-1 skin rash (*p*<0.0001 and *p*<0.0001, respectively). TTP was significantly lower in patients with *PIK3CA* mutations (*p* = 0.01) or lost PTEN (*p* = 0.002). Multivariate analysis revealed *KRAS* (Hazard Ratio [HR] 4.3, *p*<0.0001), *BRAF* mutation (HR: 5.1, *p*<0.0001), *EREG* low expression (HR: 1.6, *p* = 0.021) and absence of severe/moderate skin rash (HR: 4.0, *p*<0.0001) as independent prognostic factors for decreased TTP. Similarly, *KRAS* (HR 2.9, *p* = 0.01), *BRAF* mutation (HR: 3.0, *p* = 0.001), *EREG* low expression (HR: 1.7, *p* = 0.021), absecence of severe/moderate skin rash (HR: 3.7, *p*<0.0001) and the presence of undifferantited tumours (HR: 2.2, *p* = 0.001) were revealed as independent prognostic factors for decreased OS.

**Conclusions:**

These results underscore that *KRAS-BRAF* mutations and *EREG* expression can be used as biomarkers to further select patients undergoing anti-EGFR treatment.

## Introduction

Despite the progress made in the management of metastatic colorectal cancer (mCRC) over the last few years, the disease remains a major public health problem in the western world with an estimated 146,970 new CRC cases and 49,920 deaths for 2009 in the United States [1].

Two monoclonal antibodies targeting EGFR (anti-EGFR moAbs), both by binding to the extracellular domain, and thus, leading to inhibition of its downstream signaling, the chimeric IgG1 moAb cetuximab and the fully humanized IgG2 moAb panitumumab, have entered clinical practice in the mCRC setting and have proven to provide a modest clinical benefit in pretreated patients, either used alone or in combination with chemotherapy [2]–[5]. Nevertheless, from the beginning became clear that not all patients derive a benefit from the incorporation of these agents into the treatment combinations; indeed, non-randomized retrospective studies [6]–[11] as well as retrospective analysis of prospective randomized trials [12]–[16] demonstrated that the presence of *KRAS* mutations were predictive of resistance to anti-EGFR moAbs therapy and were associated with a worse prognosis and a shorter survival. Based on this knowledge, a primary tumor's *KRAS* mutational status is now mandatory for the treatment of metastatic disease with an anti-EGFR moAb (European Medicine Agency – EMEA-H-C-741 and H-C-558 and U.S. Food and Drug Administration - FDA Application No. (BLA) 125084 and No. (BLA) 125147).

However, not all patients with *KRAS* WT tumours benefit from anti-EGFR moAbs treatment, meaning that additional genetic determinants of resistance exist [7], [9], [17]–[19]. Indeed, from three sporadic mCRC retrospective studies [20]–[22], the *BRAF* V600E mutation has been shown to identify a subgroup (<10%) of patients that not only present resistance to anti-EGFR MoAbs therapy, but, is also characterized by particularly unfavorable prognosis regardless of treatment administration [20]–[22]. Furthermore, although not entirely clear yet, *PIK3CA*-mutant tumors seem to derive no or little benefit from anti-EGFR MoAbs treatment [20], [23]–[26].

Besides the *KRAS-BRAF-PIK3CA* mutational status, EGFR epiregulin (*EREG*) and ampiregulin (*AREG*) ligands' expression in primary CRC tumours has been shown to significantly predict clinical outcome in *KRAS* WT mCRC patients treated with cetuximab, indicating ligand-driven autocrine oncogenic EGFR signaling [27], [28]. In addition, PTEN (phosphatase and tensin homolog) protein expression, and specifically its loss, seems to be associated in a number of studies with resistance to treatment with anti-EGFR MoAbs treatment [21], [29]–[31]. Furthermore, from a clinical point of view, the only parameter which has been constantly associated with a high probability of response, prolonged progression-free survival (PFS) and median Overall Survival (mOS) to anti-EGFR moAbs treatment is the development of skin rash [2], [5], [32].

Clinical parameters seem to be inadequate for patient selection, but, biomarkers' analyses have already been incorporated in the treatment of CRC patients. The aim of the present study was to simultaneously ascertain and investigate the clinical relevance of all known biomarkers, *KRAS* exon 2, *BRAF* V600E, *PIK3CA* exon 9 and 20 mutational status in conjunction with *AREG*, *EREG* mRNA expression, PTEN immunohistochemical protein expression, as well as, skin rash development, in mCRC patients treated with cetuximab containing salvage combination chemotherapy.

## Materials and Methods

### Patient population and study design

One hundred and twelve consecutive patients, with histologically confirmed mCRC and available tumor material for molecular analysis, who were treated with cetuximab containing salvage chemotherapy at the Department of Medical Oncology, University Hospital of Heraklion (Crete, Greece) between 1/2005 - 12/2008, were enrolled. The study was approved by the Ethics and Scientific Committees of the University General Hospital of Heraklion and all patients gave their written informed consent for the use of the tissue material for translational research.

Patients' evaluation was performed at baseline and every four cycles of chemotherapy. Disease status was coded, without the knowledge of the laboratory analysis.

### Tissue selection, DNA and RNA extraction

Formalin-fixed, paraffin-embedded (FFPE) tumor sections were reviewed by a pathologist (MT) to confirm the diagnosis and define tumor-enriched areas for dissection. Ten serial sections of 5 µm thickness were stained with nuclear fast red (Sigma-Aldrich, St Louis, MO, USA) and scrape dissection under a binocular microscope was performed for samples with ≥80% tumor cells; for samples with <80% malignant cells, microdissection with the piezoelectric Eppendorf microdissector (Eppendorf, Hamburg, Germany) was performed. DNA extraction was performed with the use of the Epicentre® Biotechnologies MasterPure™ Complete DNA and RNA Purification Kit according to the manufacturer's instructions (Epicentre, Madison, WI, USA) after the isolated cancer cells were lysed in buffer containing Proteinase K at 60°C for 72 h. For RNA extraction, cancer cells were re-suspended in 400 µl RNA lysis buffer supplemented with 300 mg proteinase K (QIAGEN, Valencia, CA, USA) and incubated at 60°C for 16 hours until the tissue was completely solubilized. RNA was purified by Trizol LS (Invitrogen, Carlsbad,CA,USA) and, subsequently, treated with DNase (DNΑ- free, Ambion, Austin, TX, U.S.A.) in order to avoid genomic DNA contamination and stored at -80°C until used.

### *KRAS* and PIK3CA mutational analysis

*KRAS* and PIK3CA mutational analysis was performed by Sanger sequencing after PCR amplification of KRAS exon 2 and PIK3CA exons 9 and 20. PCR conditions with primers sets which have been previously reported [22].

### *BRAF* mutational analysis

The V600E *BRAF* mutation was detected by real-time PCR using the allelic discrimination method as previously described [33], [34]. In brief, the DNA extracted from tumoral cells was amplified with the use of a set of primers and two hydrolysis probes in the ABI PRISM 7900T Sequence Detection System (AB; Applied Biosystems, Forest City; CA; USA). The two hydrolysis probes were labeled at 5 with VIC and FAM fluorophores reporters for the wt and the mutant allele, respectively. The SDS 2.3 software was used for the analysis of the results.

### *AREG* and *EREG* mRNA expression

The SuperScript III Reverse Transcriptase (Invitrogen, Carlsbad, CA, U.S.A.) was used to prepare cDNA from 50 ng of total RNA for each gene analyzed as previously described [35]. Relative cDNA quantification for *AREG, EREG* and both *β-actin* and *PGK* as internal reference genes was done using the ABI Prism 7900HT Sequence Detection System (AB), as described previously [35]. The primers and probe sets were designed using Primer Express 2.0 Software (AB), according to the Ref Seq NM_001657.2 for *AREG* and NM 001432.2 for *EREG* (http://www.ncbi.nlm.nih.gov/LocusLink). The sequence of the primers and 5′ labeled fluorescent reporter dye (6FAM) probes for all reference and target genes are shown in **Table 1** **.**

**Table 1:** Sequence of the primers and probes of all references and target genes.

Gene	Forward Primer	5′-labeled (FAM) probe	Reverse Primer
***β-actin***	5′-GGC ACC CAG CAC AAT GAA G-3′	**5′** TCA AGA TCA TTG CTC CTC CTG AGC GC**--3**	**’**5′-GCC GAT CCA CAC GGA GTA CT-3′
***PGK***	5′- GGCTGGATGGGCTTGGA –3′	5-TGTGGTCCTGAAAGCAGCAAGAAGTATGC -3′	5′-TCTGCTTAGCCCGAGTGACA-3
***AREG***	5′- GTGGTGCTGTCGCTCTTGATAC -3′	5- CGGCTCAGGCCATTATGCTGCTG-3′	5′-AGAGTAGGTGTCATTGAGGTCCAAT-3′
***EREG***	5′- TGCATCTATCTGGTGGACATGAG -3′	5-AAAACTACTGCAGGTGTGAAGTGGT-3′	5′-AGTGTTCACATCGGACACCAGTA –3′

Relative gene expression quantification was performed according to the comparative Ct method using *β-actin* and *PGK* as endogenous controls and commercial RNA controls (Stratagene, La Jolla, CA, USA) as calibrators. Final results were determined as follows: 2^-(ΔCt sample-ΔCt calibrator)^, where ΔC_T_ values of the calibrator and sample were determined by subtracting the C_T_ value of the target gene from the mean value of both reference genes. In all experiments, only triplicates with a standard deviation (SD) of the Ct value <0.25 were accepted. In addition, genomic DNA contamination of each sample has been excluded by non-reverse transcription of RNA [35].

### PTEN protein expression

Three- to 4- *µ*m tumor tissue sections of paraffin-embedded specimens from each patient were selected for PTEN IHC staining using the 17.A mouse monoclonal antibody (1∶25 dilution, Neomarkers; ThermoFisher Scientific Inc, Fremont, CA), as previously described [28], [36]. After deparaffinization and hydration of sections, antigens were unmasked by heat in EDTA buffer. Immunostaining was performed using the UltraVision LP Large Volume Detection System AP Polymer (Thermo Scientific, Waltham, MA, USA). Negative control slides were prepared by omitting the primary antibody. Prostate cancers and endothelial cells were used as external and internal positive controls, respectively.

PTEN staining was mainly cytoplasmatic. As previously described [28], intensity was scored according to a four-tier system: 0, no staining; 1, weak; 2, moderate; and 3, strong. One, two or three additional points were attributed if the percentage of positive was <25%, 25–50% or >50%, respectively. The specimens with a cumulative score of ≥4 were characterized as positive [28].

### Study Design and Statistical analysis

The present study was a retrospective analysis aiming to explore the predictive value of extensive biomarkers analysis in the outcome of patients with mCRC treated with cetuximab plus chemotherapy as salvage treatment. All available biopsies of the primary tumor with more than 100 cells per section were included in the analysis. RT-qPCR analysis yielded values that were expressed as ratios between two absolute measurements (gene of interest: mean of internal reference genes). CART analysis has been used for the estimation of the cut-off points of *AREG* and *EREG* mRNA expression, in order to classify cases into groups of a dependent (TTP and mOS) variable. Samples with mRNA expression above or equal to the cut-off point were considered as samples with high expression, while those with value below the median as samples with low expression. Associations between *KRAS, BRAF*, *PIK3CA* mutation status, *AREG* and *EREG* mRNA expression and PTEN IHC expression with baseline characteristics were assessed using the Fisher's exact test for categorical variables or logistic regression for continuous variables. Spearman's exact test was used to evaluate the correlation between *AREG* and *EREG* mRNA expression. Time to tumour progression (TTP) and overall survival (OS) were measured from the date of the cetuximab containing treatment line initiation to the first radiographic documentation of disease progression or death, respectively. Kaplan–Meier curves were used to describe the proportion of patients who remained free of events over the follow-up period. Associations between prognostic factors and TTP or OS were examined using Cox proportional hazards regression models. All reported *p*-values are two-sided and not adjusted for multiple testing.

## Results

### Patient demographics

The mutational status for *KRAS* exon 2, *BRAF* exon 15, and *PIK3CA* exons 9 and 20 was determined in all 112 consecutive patients with mCRC whereas. *AREG* and *EREG* mRNA expression was determined in 106 and 105 patients for whom tumour material was available respectively, while PTEN expression was evaluated in 106 patients. All patients were treated with cetuximab in combination with chemotherapy (73% in combination with Irinotecan, 27% with Oxaliplatin) as salvage treatment (Table 2). Sixty-six (59%) patients had received the treatment in the 2^nd^ line setting and the remaining 46 (41%) as 3^rd^ line treatment. There was no patient who received the anti-EGFR moAbs in the 1^st^ line setting. Disease characteristics were typical for mCRC in the western world; the patients' median age was 66 years and 60% of them were male **(** **Table 2** **)**. The median PFS from 1^st^ line treatment was 8.9 months (95% CI 8.1-9.9) and the median time from relapse to previous treatment line until the cetuximab administration was 1.1 months (95% CI 0.7–1.8).

**Table 2:** Patients′ and tumors′ characteristics.

Feature	Ν	%
	112	
**Median Age** (Range)	66(23–83)	
≤70 years	76	78
>70 years	36	32
**Gender**		
Male	68	60
Female	44	40
**Stage at diagnosis**		
I-III	61	54
IV	51	46
**Tumor Location**		
Colon	83	74
Rectum	29	26
**Tumor differentiation**		
Well moderate	66	59
Undifferentiated	46	41
**Mucinous Features**		
Yes	18	16
No	94	84
**Cetuximab administration line**		
2nd	66	59
3rd	46	41
**Chemotherapy administered with Cetuximab**		
Irinotecan-based	82	73
Oxaliplatin-based	30	27

### Mutational status and expression values results

**Figure 1:**
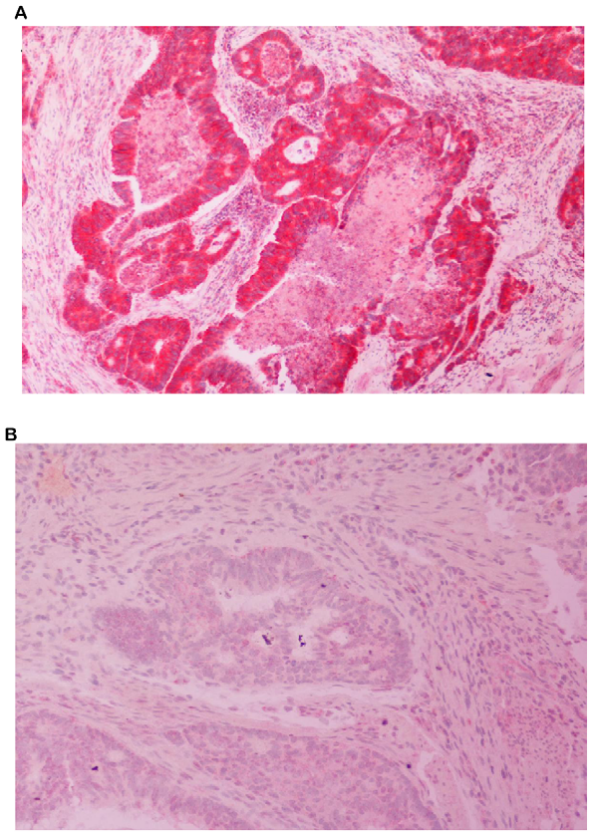
Assessment of PTEN expression by immunohistochemistry. Panel A: Sample of a moderate differentiated adenocarcinoma of the colon scored as PTEN positive (x100) Panel A: Sample of a moderate differentiated adenocarcinoma of the colon scored as PTEN negative (x100).

*KRAS* mutations were detected in 37 (33%), *BRAF* mutations in eight (7.2%) and *PIK3CA* mutations in 11 (9.8%, 8 in exon 9 and 3 in exon 20) primary tumours, respectively. *KRAS* and *BRAF* mutations were mutually exclusive, whereas, three tumours carried both *KRAS* and *PIK3CA* mutations. *AREG* and *EREG* were overexpressed in 48 (45%) and 51 (49%) patients, respectively, whereas, PTEN was scored as negative (i.e. loss of function) in 21 (19.8%) patients (Figures 1A and 1B). When *PIK3CA* mutations and PTEN expression were analyzed together, activation of the pathway (defined as loss of PTEN or *PIKECA* mutation) was detected in 25 (23.5%) patients. A trend for decreased incidence of *KRAS* mutations in rectal tumors was observed (*p* = 0.097) since 31 of the 83 (37%) tumours located at the colon and six of the 29 (20%) tumours located at the rectum harbored a *KRAS* mutation. There was no correlation between the presence of *KRAS* mutations with the patients' gender, age (>70 years old versus ≤70 years old), stage at diagnosis, histological grade, mucinous status, PTEN loss and *AREG*-*EREG* expression (all *p*-values >0.05). Also, a statistically significant correlation was observed between the presence of *BRAF* mutations and the histological grade (well/moderate versus undifferentiated) (*p* = 0.049) and *EREG* mRNA downregulation (*p* = 0.013). There was no correlation between the presence of *BRAF* and *PIK3CA* mutations with the patients' gender, age (>70 years old versus ≤70 years old), stage at diagnosis, tumour location, mucinous status, PTEN loss and *AREG* expression (in both cases all *p*-values >0.05).

### Impact of mutational status and expression values on the outcome of salvage cetuximab therapy

#### Results in the whole patients' population (Table 3)

**Tables 3** **and** **4** summarize the impact of genetic alterations on the outcome of cetuximab-containing salvage treatment. The median TTP of the whole group of patients was 4.9 months (95% CI 4.1–5.7) and the corresponding median overall survival (OS) 14.5 months (95% CI 10.0–18.9). TTP and OS were significantly lower among patients whose tumours carried *KRAS* mutations (3.1 vs. 6.4 months, *p* = 0.001 and 10.6 vs. 16.3 months, *p* = 0.026, respectively) **(** **Figure 2A and 2B** **).** Similarly, TTP and OS were significantly lower among patients whose tumours carried *BRAF* mutations (2.1 vs. 5.2 months, *p* = 0.001 and 4.3 vs. 15.1 months, *p*<0.0001, respectively) **(** **Figure 3** **and** **4** **).** There was no significant correlation in terms of TTP according to *PIK3CA* mutational status or PTEN expression in all treated patients (4.9 vs. 5. 7 months, *p* = 0.427 and 5.2 vs. 6.03. months, *p* = 0.102, respectively) **(** **Figure 4A and 4B** **)**; similarly, there was no difference in terms of median OS between patients with *PIK3CA* mutant (13.6 months) and wt (15.0 months) primary tumours (*p* = 0.44; **Figure 5A**), as well as between patients with lost (14.3 months) or normal (15.1 months) PTEN function (p = 0.82; **Figure 5B**). Nevertheless, when *PIK3CA* mutational status and PTEN expression were taken into consideration together, activation of the pathway through *PIK3CA* mutations and/or PTEN loss was correlated with a trend for decreased TTP in all patients (3.8 vs. 5.0 months, *p* = 0.051) **(** **Figure 4E** **)**, while no difference was observed in the median OS (13.9 vs. 14.5 months, *p* = 0.878) **(** **Figure 5E** **)**.

**Figure 2:**
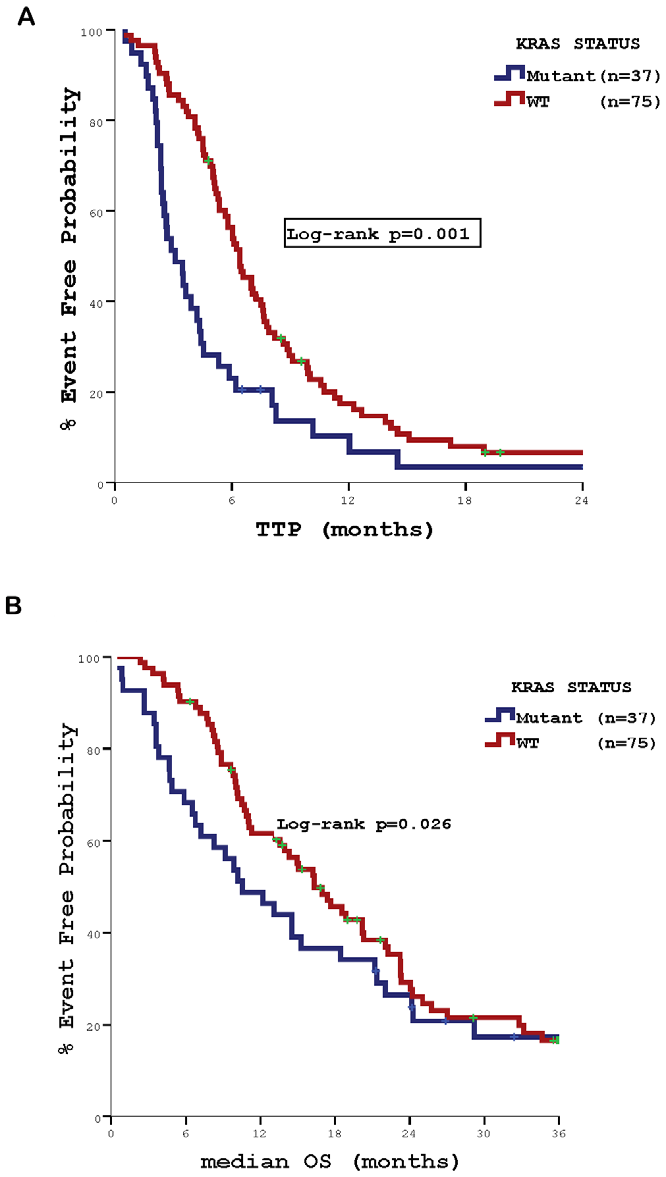
Patients' outcome according to *KRAS* mutations status. Panel A: Time to Tumor Progression (TTP) Panel B: Median Overall Survival (OS).

**Figure 3:**
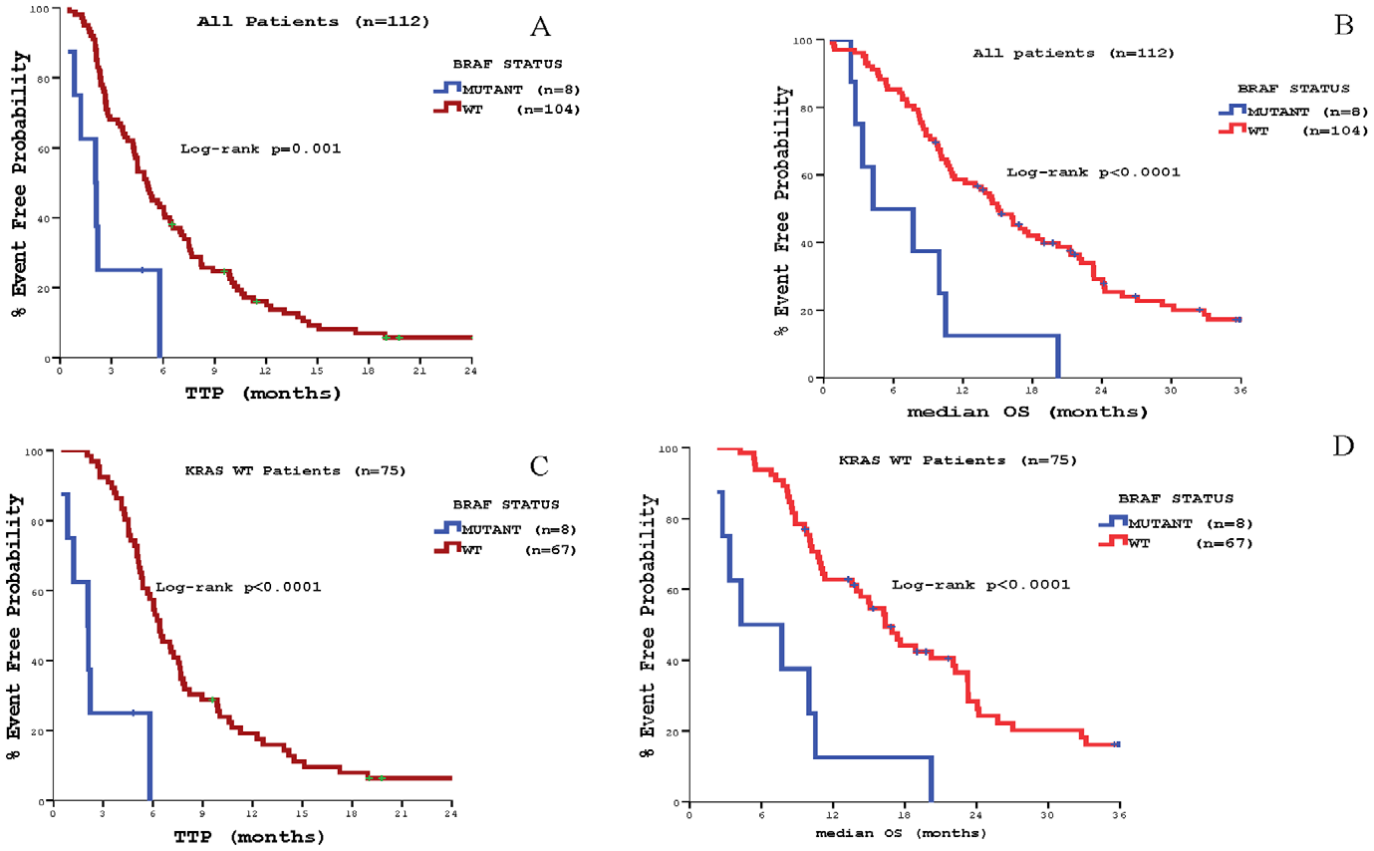
Patients' outcome according to *BRAF* mutations status. Panel A: Time to Tumor Progression (TTP) in the whole patients' population. Panel B: Median Overall Survival (OS) in the whole patients' population Panel C: Time to Tumor Progression (TTP) in patients with *KRAS* wt primary tumors. Panel D: Median Overall Survival (OS) in patients with *KRAS* wt primary tumors.

**Figure 4:**
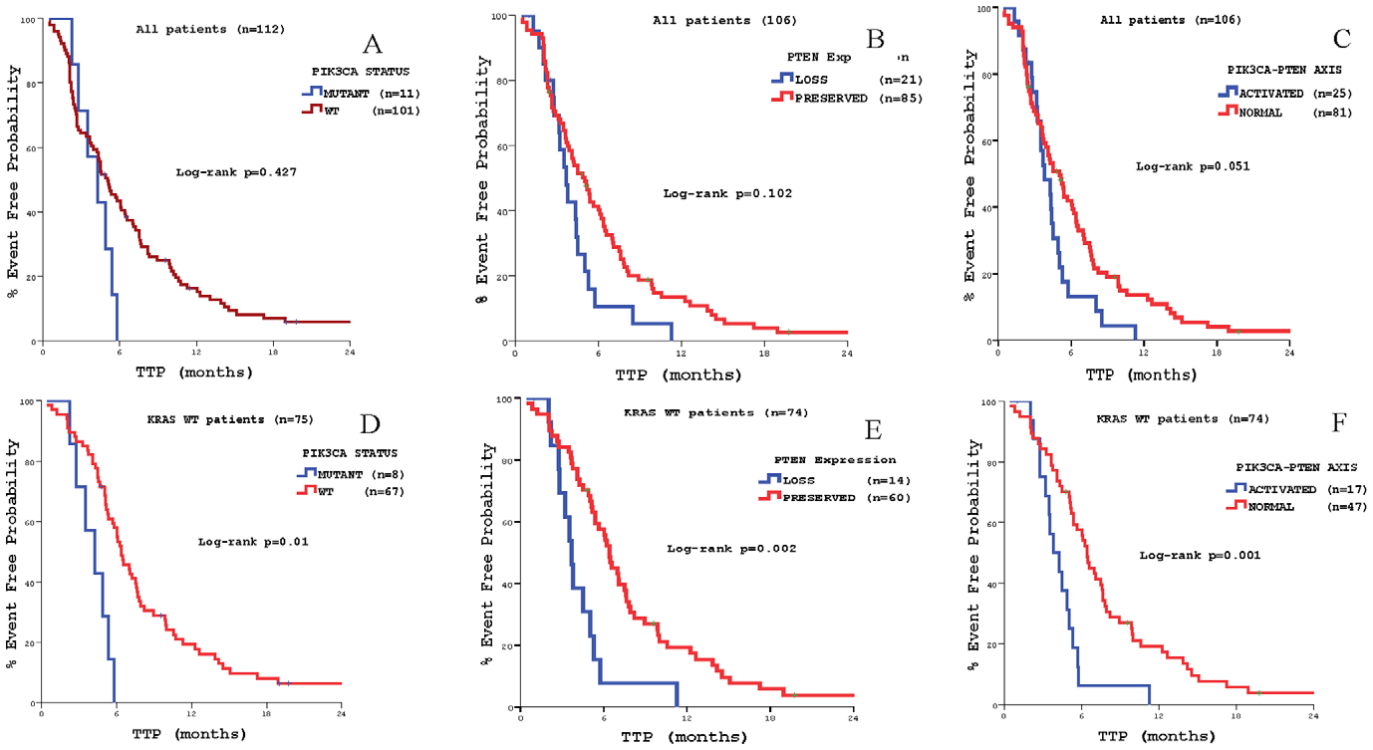
Time to Tumor Progression (TTP according to *PIK3CA mutations* status and PTEN expression. Panel A: according to *PIK3CA* mutations status in the whole patients' population. Panel B: according to PTEN expression in the whole patients' population. Panel C: according to PIK3-PTEN axis activation status (*PIK3CA* mutations status and PTEN expression) in the whole patients' population. Panel D: according to *PIK3CA* mutations status in patients with *KRAS* wt primary tumors. Panel E: according to PTEN expression in patients with *KRAS* wt primary tumors. Panel F: according to PIK3-PTEN axis activation status (*PIK3CA* mutations status and PTEN expression) in patients with *KRAS* wt primary tumors.

**Figure 5:**
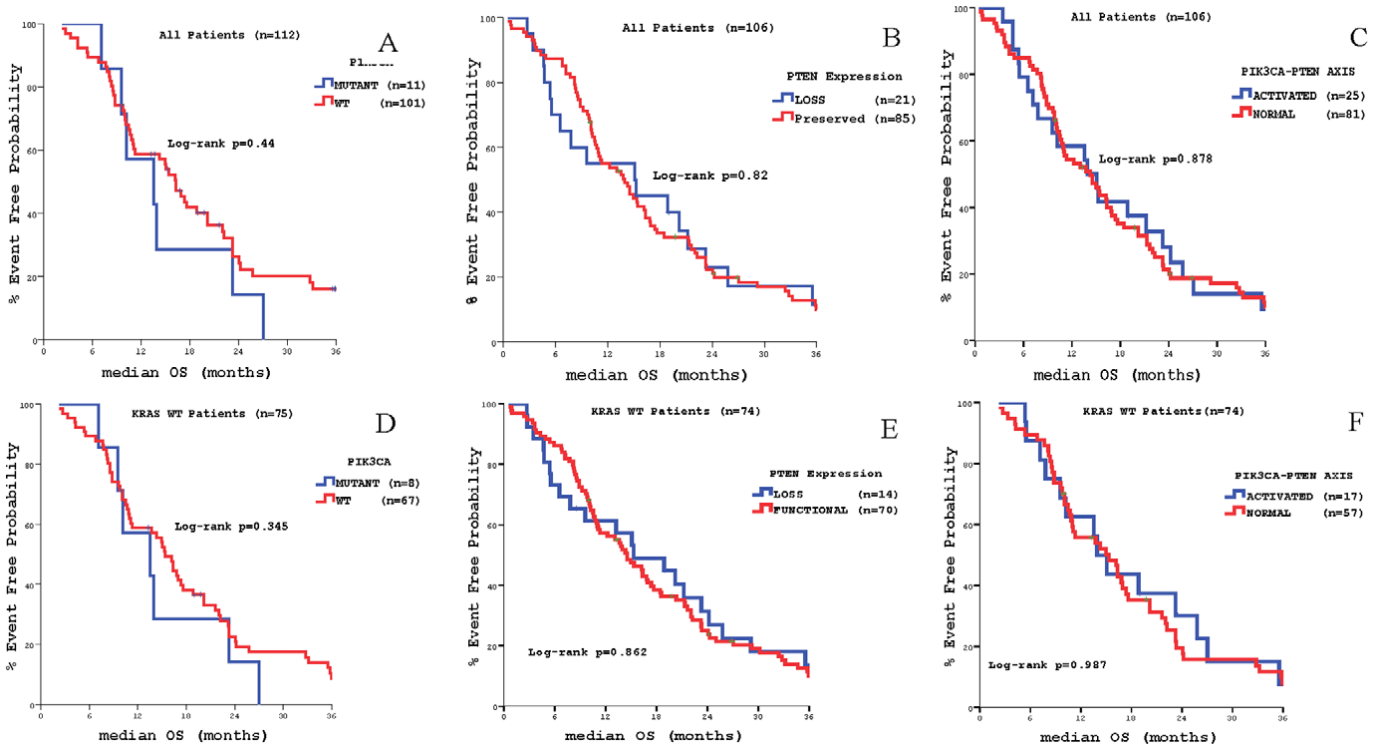
Median Overall Survival (OS) according to *PIK3CA mutations* status and PTEN expression. Panel A: according to *PIK3CA* mutations status in the whole patients' population. Panel B: according to PTEN expression in the whole patients' population. Panel C: according to PIK3-PTEN axis activation status (*PIK3CA* mutations status and PTEN expression) in the whole patients' population. Panel D: according to *PIK3CA* mutations status in patients with *KRAS* wt primary tumors. Panel E: according to PTEN expression in patients with *KRAS* wt primary tumors. Panel F: according to PIK3-PTEN axis activation status (*PIK3CA* mutations status and PTEN expression) in patients with *KRAS* wt primary tumors.

**Table 3:** TTP and OS to the ≥2^nd^ line cetuximab-containing treatment according to *KRAS*, *BRAF*, *PIK3CA* mutations status, PTEN protein expression, *AREG* and *EREG* mRNA expression and grade of skin rash in the whole patient′ population.

				Time to Tumor Progression (months)			Overall survival (months)		
All patients n = 112				4.9 months (95% CI 4.1–5.7)			14.5 months (95% CI 10.0–18.9).		
Feature	Patients′ population(No of patients)			Median (months)(95% CI*)	HR#(95% CI)	*p* value	Median (months)(95% CI)	HR(95% CI)	*p* value
*KRAS* status	n = 112	Mutant	(n = 37)	3.1 (2.0–4.2)	3.3 (2.4–5.1)	0.001	10.6 (5. 7–15.5)	2.2 (1.7–2.8)	0.026
		WT∧	(n = 75)	6.4 (5.4–7.4)			16.3(12.7–19.6)		
*BRAF* status	n = 112	Mutant	(n = 8)	2.1 (0.8–3.3)	4.9 (2.2–10.9)	0.001	4.3 (0.3–10.3)	3.6 (1.7–7.5)	<0.0001
		WT∧	(n = 104)	5.2 (4.3–6.1)			15.1 (12.2–17.9)		
*PIK3CA* status	n = 112	Mutant	(n = 11)	4.9 (2.9–6.9)	1.9 (0.9–4.1)	0.427	13.6 (4.9–19.2)	1.3 (0.7–2.9)	0.44
		WT∧	(n = 101)	5.7 (4.8–6.8)			15.0 (13.2–22.2)		
PTEN expression	n = 106	Loss	(n = 21)	5.2 (4.1–6.3)	1.7 (0.97–2.8)	0.102	14.3 (2.6–18.8)	1.1 (0.6–1.8)	0.82
		Preserved	(n = 85)	6.0 (4.9–7.2)			15.1 (9.8–24.3)		
*PIK3CA-*PTEN axis	n = 106	Activated	(n = 25)	3.8 (2.7–4.9)	1.6 (1.0–2.6)	0.051	13.9 (7.8–20.0)	1.1 (0.7–1.7)	0.878
		Normal	(n = 81)	5.0 (3.9–6.1)			14.5 (9.6–19.4)		
*AREG* expression	n = 106	Downregulated	(n = 58)	3.8 (2.7–4.9)	1.7 (1.1–3.2)	0.018	10.7 (9.5–11.9)	1.7 (1.1–2.6)	0.013
		Overexpressed	(n = 48)	5.0 (3.9–6.1)			20.2 (12.8–27.6)		
*EREG* expression	n = 105	Downregulated	(n = 54)	6.1 (3.9–8.3)	2.1 (1.3–3.1)	0.002	10.7 (9.5–11.9)	1.8 (1.2–2.8)	0.004
		Overexpressed	(n = 51)	3.6 (2.00–5.3)			17.6 (12.6–22.7)		
Skin rash	n = 112	None	(n = 24)	2.3 (1.9–2.7)	5.1 (2.9–9.1)$	<0.0001$	4.9 (2.8–6. 9)	5.3 (3.0–9.4)$	<0.0001$
		Grade 1	(n = 40)	4.5 (3.3–5.7)	2.5 (1.5-4.0)^@^	<0.0001^@^	13.2 (8.9–17.5)	2.2 (1.4-3.7)^@^	<0.0001^@^
		Grade 2–3	(n = 48)	7.5 (6.0–9.0)			24.1 (21.4–26.7)		
*KRAS* -*BRAF* -*AREG* genotype	*KRAS* or *BRAF* mutant *AREG* downregulated		(n = 25)	2.3 (1.8–2.9)	7.0(3.8–12.9)&	<0.0001&	9.9 (6.1–13.7)	3.1(2.1–3.6) &	0.001&
	*KRAS* or *BRAF* mutant *AREG* overexpressed		(n = 14)	3.1 (2.1–4.1)	5.1 (2.64–10.0)∞	<0.0001∞	10.2 (3.7–16.6)	2.2 (1.3–3.8)∞	0.017∞
	*KRAS* or *BRAF* WT *AREG* downregulated		(n = 33)	4.6 (3.8–5.4)	2.5 (1.5–4.2) £	<0.0001£	10.2 (8.8–11.6)	2.0 (1.1–3.8)£	0.019£
	*KRAS* or *BRAF* WT *AREG* overexpressed		(n = 34)	9.9 (7.6–12.2)			23.3 (21.3–25.2)		
*KRAS* -*BRAF* -*EREG* genotype	*KRAS* or *BRAF* mutant *EREG* downregulated		(n = 19)	2.2 (1.9–2.5)	16.8(11.8–31.4)&	<0.0001&	9.2 (3.2–15.1)	3.5(2.5–4.4)&	<0.0001&
	*KRAS* or *BRAF* mutant *EREG* overexpressed		(n = 17)	3.5 (2.4–4.6)	6.8 (3.4–13.8)∞	<0.0001∞	10.1 (5.6–14.7)	2.2 (1.2–3.9) ∞	0.013∞
	*KRAS* or *BRAF* WT *EREG* downregulated		(n = 35)	5.0 (4.3–5.8)	2.6 (1.5–4.3)£	<0.0001£	10.2 (9.1–11.3)	2.1 (1.1–3.8)£	0.015£
	*KRAS* or *BRAF* WT *EREG* overexpressed		(n = 34)	8.2 (5.3–11.1)			23.2 (17.8–28.7)		

*CI: Confidence Interval,

# HR: Hazard Ration,

∧ WT: Wild Type,

$ Skin rash grade 2–3 vs. none,

@ Skin rash grade 2–3 vs. grade 1,

& *KRAS* or *BRAF* WT and *EREG* overexpressed vs. *KRAS* or *BRAF* mutant and *EREG* downregulated,

∞ *KRAS* or *BRAF* WT and *EREG* overexpressed vs. *KRAS* or *BRAF* mutant and EREG overexpressed,

£ *KRAS* or *BRAF* WT and *EREG* overexpressed vs. *KRAS* or *BRAF* WT *EREG* downregulated.

**Table 4:** TTP and OS to the ≥2^nd^ line cetuximab-containing treatment according to *KRAS*, *BRAF*, *PIK3CA* mutations status, PTEN protein expression, *AREG* and *EREG* mRNA expression and grade of skin rash in the *KRAS* WT patients′ population.

						Time to Tumor Progression (months)			Overall survival (months)		
*KRAS* WT patients n = 75						6.4 months (95% CI 5.4–7.4)			16.3 months (95% CI 12.7–19.6).		
Feature	Patients′ population(No of patients)					Median (months)(95% CI*)	HR#(95% CI)	*p* value	Median (months)(95% CI)	HR(95% CI)	*p* value
*BRAF* status	n = 75		Mutant		(n = 8)	2.1 (0.2–3.4)	9.5 (3.9–23.3)	<0.0001	4.3 (0.2–10.3)	4.6 (2.1–10.0)	<0.0001
			WT∧		(n = 67)	6.4 (5.3–7.5)			16.3 (13.6–19.1)		
*PIK3CA* status	n = 75		Mutant		(n = 8)	4.3(2.3–6.2)	3.3 (1.4–7.7)	0.01	13.5 (4.9–18.8)	1.5 (0.8–3.3)	0.345
			WT∧		(n = 67)	6.4 (5.3–7.4)			16.3 (4.9–18.8)		
PTEN expression	n = 74		Loss		(n = 14)	3.7 (2.9–4.5)	2.7 (1.4–5.1)	0.002	15.3 (6.2–22.8)	1.1 (0.7–2.0)	0.862
			Preserved		(n = 60)	5.0 (4.0–6.0)			14.5 (11.8–21.3)		
*PIK3CA-*PTEN axis	n = 74		Activated		(n = 17)	3.8 (2.4–5.2)	2.9 (1.6–5.3)	0.001	13.9 (11.0–18.9)	1.1 (0.7–1.8)	0.987
			Normal		(n = 57)	6.4 (5.7–7.0)			16.2 (13.3–19.1)		
*AREG* expression	n = 75		Downregulated		(n = 39)	4.3 (2.8–5.7)	2.0 (1.3–2.5)	0.021	10.7 (11.9–18.2)	2.2 (1.3–3.8)	0.004
			Overexpressed		(n = 36)	5.8 (4.0–7.6)			23.2 (18.5–27.9)		
*EREG* expression	n = 75		Downregulated		(n = 39)	3.8 (1.6–5.9)	2.3 (1.4–3.9)	0.001	10.5 (9.4–11.6)	2.9 (1.7–5.0)	<0.0001
			Overexpressed		(n = 36)	7.0 (4.8–9.2)			20.2 (13.4–27.0)		

*CI: Confidence Interval,

# HR: Hazard Ration,

∧ WT: Wild Type,

$ Skin rash grade 2–3 vs. none,

@ Skin rash grade 2–3 vs. grade 1,

& *KRAS* or *BRAF* WT and *EREG* overexpressed vs. *KRAS* or *BRAF* mutant and *EREG* downregulated,

∞ *KRAS* or *BRAF* WT and *EREG* overexpressed vs. *KRAS* or *BRAF* mutant and EREG overexpressed,

£ *KRAS* or *BRAF* WT and *EREG* overexpressed vs. *KRAS* or *BRAF* WT *EREG* downregulated.

**Figure 6:**
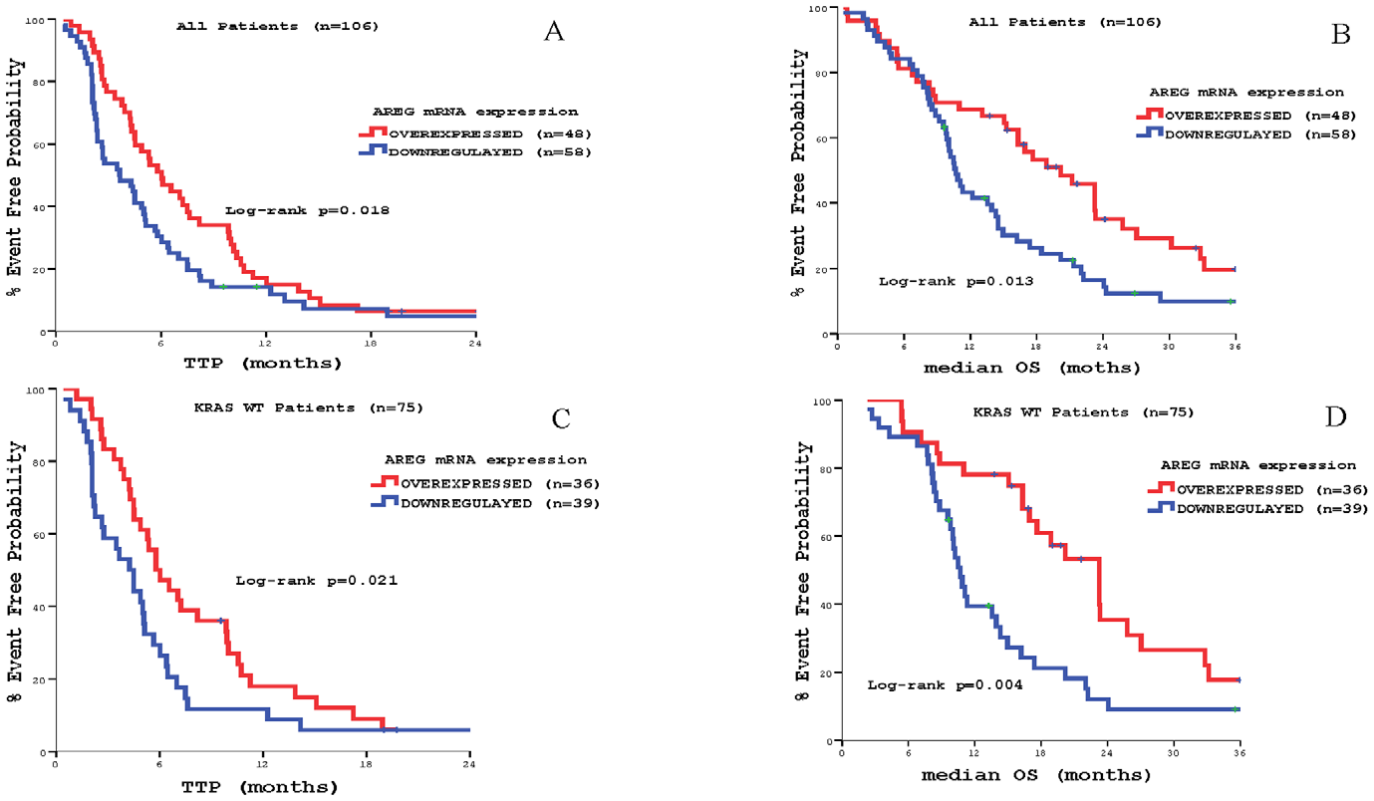
Patients' outcome according to *AREG* mRNA expression. Panel A: Time to Tumor Progression (TTP) in the whole patients' population. Panel B: Median Overall Survival (OS) in the whole patients' population Panel C: Time to Tumor Progression (TTP) in patients with *KRAS* wt primary tumors. Panel D: Median Overall Survival (OS) in patients with *KRAS* wt primary tumors.

**Figure 7:**
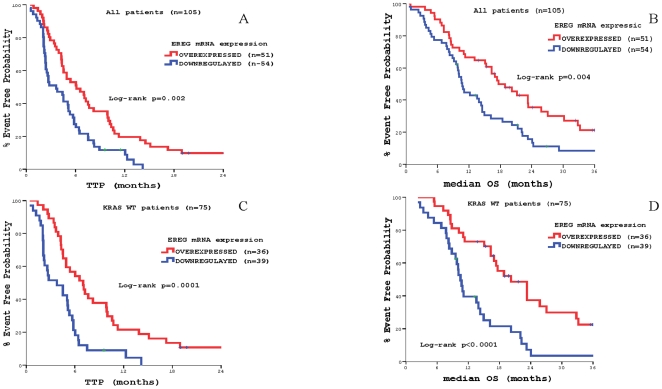
Patients' outcome according to *EREG* mRNA expression. Panel A: Time to Tumor Progression (TTP) in the whole patients' population. Panel B: Median Overall Survival (OS) in the whole patients' population Panel C: Time to Tumor Progression (TTP) in patients with *KRAS* wt primary tumors. Panel D: Median Overall Survival (OS) in patients with *KRAS* wt primary tumors.

A highly significant correlation between *AREG* and *EREG* mRNA expression was observed (Spearman ρ^2^ = 0.736, *p<0.001*). In the whole group of patients, *AREG* mRNA overexpression was significantly correlated with increased TTP and OS (5.0 vs. 3.8 months, *p* = 0.018 and 20.2 vs. 10.7 months, *p* = 0.013, respectively]) **(** **Figures 6A and 6B** **)**. Furthermore, *EREG* mRNA overexpression was also correlated significantly with increased TTP and OS (6.1 vs. 3.6 months, *p* = 0.002 and 17.6 vs. 10.7 months, *p* = 0.004, respectively) **(** **Figures 7A and 7B** **)**.

**Figure 8:**
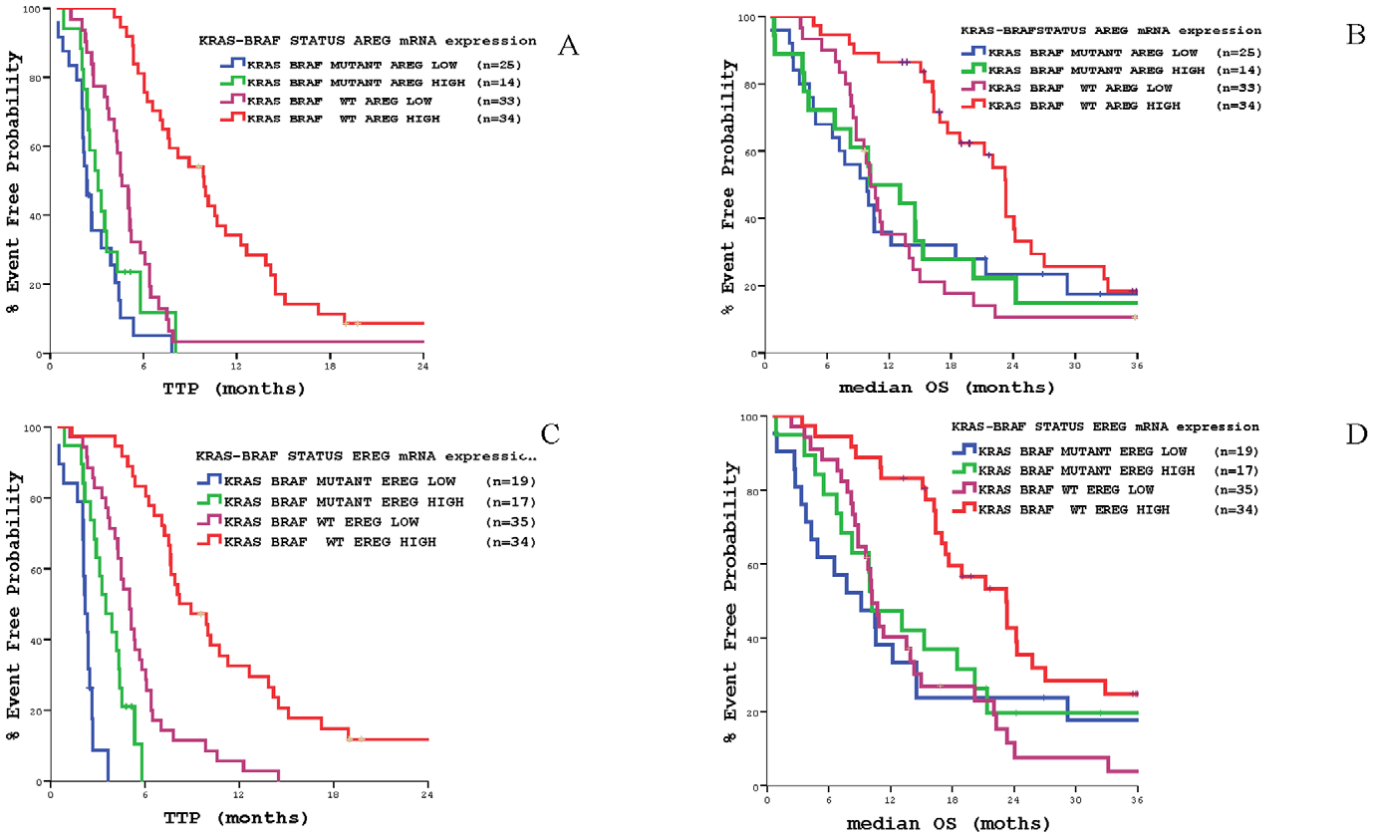
Patients' outcome according to *KRAS-BRAF* mutations status *and AREG or EREG* mRNA expression. **Panel** A: Time to Tumor Progression (TTP) according to *KRAS-BRAF* mutations status and *AREG* mRNA expression. Panel B: Median Overall Survival (OS) according to *KRAS-BRAF* mutations status and *AREG* mRNA expression. Panel C: Time to Tumor Progression (TTP) according to *KRAS-BRAF* mutations status and *EREG* mRNA. Panel D Median Overall Survival (OS) according to *KRAS-BRAF* mutations status and *EREG* mRNA.

**Table 3** and **Figures 8A and 8B** demonstrate the differences in TTP and OS according to *KRAS–BRAF* mutational status and *AREG* expression. It is shown that the *KRAS-BRAF* WT and *AREG* overexpression profile was correlated significantly with increased TTP and OS compared with any other combination. Similarly, **Figures 8C and 8D** **and** **Table 3** illustrate the differences in TTP and OS according to *KRAS–BRAF* mutational status and *EREG* expression; again, the *KRAS-BRAF* WT and EREG overexpression profile was correlated significantly with increased TTP and OS compared with any other combination.

**Figure 9:**
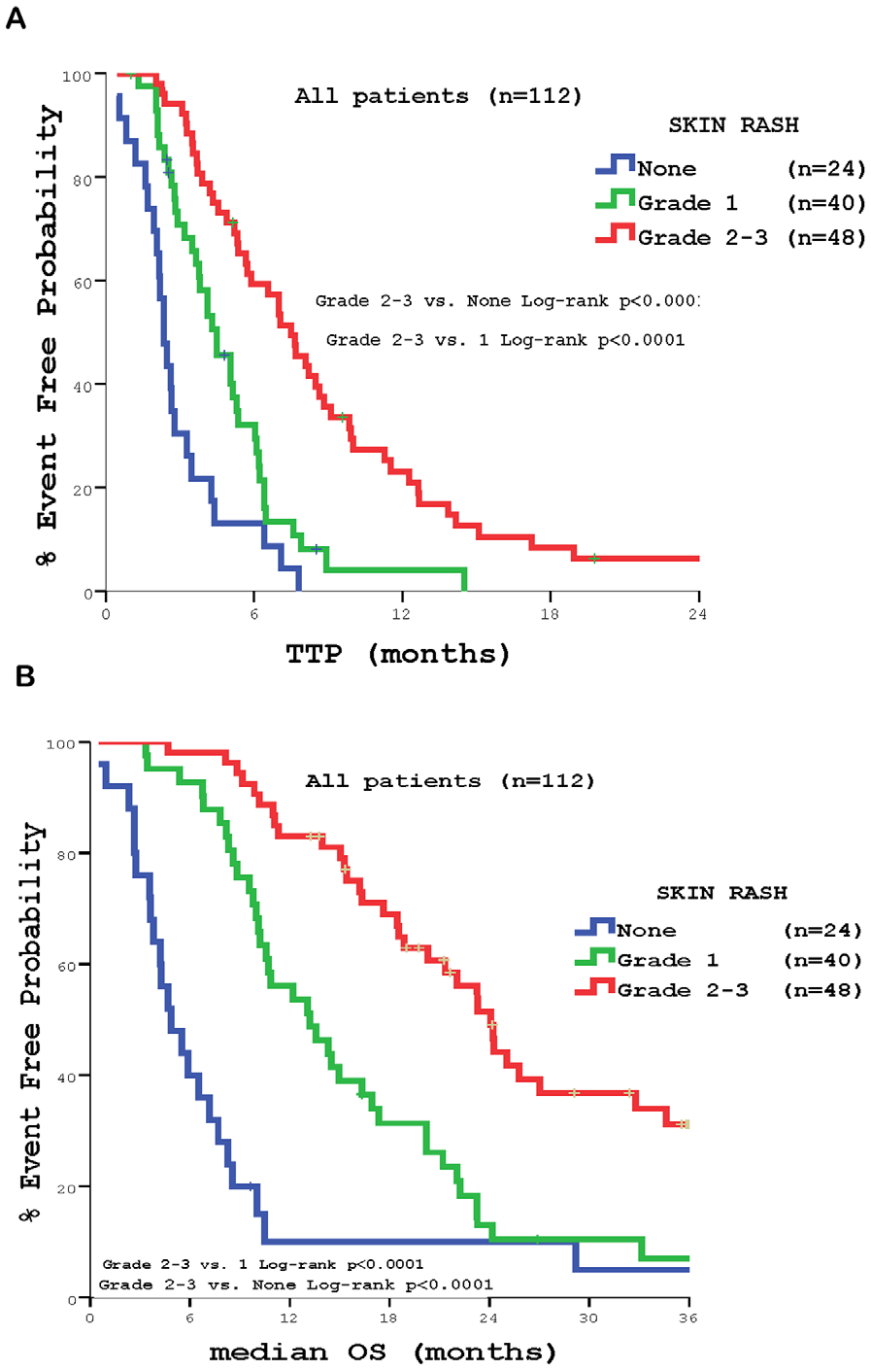
Patients' outcome according to severity of skin rash during the cetuximab administration. Panel A: Time to Tumor Progression (TTP) according to the worst skin rash grade developed during the treatment with cetuximab + chemotherapy. Panel B: Median Overall Survival (OS) according to the worst skin rash grade developed during the treatment with cetuximab + chemotherapy.

Finally, we correlated the impact of cetuximab induced skin rash with treatment outcome. Patients with severe or moderate (grade 2–3) skin rash presented significantly higher TTP (7.5 months) in comparison with those with mild (grade 1) (4.5 months; *p*<0.0001) and no skin rash (2.3 months, *p*<0.0001), as well as increased OS (24.1 vs. 13.2 months, *p*<0.0001, and vs. 4.9 months, *p*<0.0001) **(** **Table 3** **and** **Figures 9A and 9B** **)**.

#### Results in the *KRAS* WT patients' population (Table 4)

When only *KRAS* WT cases were analyzed patients whose tumours carried the *BRAF* mutation had even more significantly lower TTP and OS (TTP: 2.1 vs. 6.4 months, *p*<0.0001; OS: 4.3 vs. 16.3 months, *p* <0.0001) **(** **Figure 3C and 3D** **)** compared with the results in the whole population. In addition, when only the *KRAS* WT cases were considered, decreased TTP was significantly associated with the presence of *PIK3CA* mutation (4.3 vs. 6.4 months, *p* = 0.01) **(** **Figure 4C** **)** and PTEN downregulation (3.7 vs. 5.0 months, *p* = 0.002) **(** **Figure 4D** **)**. Nevertheless, in this particular group of patients with *KRAS* WT tumors, no significant correlation was found in the median OS between patients with or without *PIK3CA* mutations (13.5 vs. 16.3 months, respectively; p = 0.345) or those with downregulated or functional PTEN (15.3 vs. 14.5 months, respectively; p = 0.862) **(** **Figures 5C and 5D** **)**. But, in *KRAS* WT patients when *PIK3CA* mutational status and PTEN expression were taken into consideration together, a significantly decreased TTP was observed with the activation of the pathway through *PIK3CA* mutations and/or PTEN loss, compared with its inactivated presence with wt *PIK3CA* and/or functional PTEN (3.8 vs. 6.4 months, *p* = 0.001) **(** **Figure 4F** **)**; conversely, such a correlation could not be revealed in terms of median OS (13.9 vs. 16.2 months; *p* = 0.987) **(** **Figure 5F** **)**.

In *KRAS* WT patients, *AREG* mRNA overexpression was significantly correlated with increased TTP and OS (5.8 vs. 4.3 months, *p* = 0.021 and 23.2 vs. 10.7 months, *p* = 0.004, respectively) **(** **Figures 6C and 6D** **)**, as well as, *EREG* mRNA overexpression (7.0 vs. 3.8 months, *p* = 0.0001 and 20.2 vs. 10.5 months, *p*<0.0001, respectively) **(** **Figures 7C and 7D** **)**.

### Univariate and Multivariate analysis

As far as TTP was concerned, the univariate analysis **(** **Table 3** **and** **4** **)** demonstrated significant associations with: i) *KRAS* mutations (*p* = 0.001); ii) *BRAF* mutations (*p* = 0.001); iii) *AREG* mRNA expression (*p* = 0.018); iv) *EREG* mRNA expression (*p* = 0.002) and v) the development of moderate severe skin rash (*p*<0.0001). In addition, TTP in *KRAS* wt patients was significantly correlated with *PIK3CA* mutation (*p* = 0.01), PTEN expression (*p* = 0.002) and the *PIK3CA*-PTEN axis activation (*p* = 0.001). As far as OS was concerned the univariate analysis **(** **Table 3** **and** **4** **)** demonstrated significant associations with: i) *KRAS* mutations (*p* = 0.026); ii) *BRAF* mutations (*p*<0.0001); iii) *AREG* mRNA expression (*p* = 0.013); iv) *EREG* mRNA expression (*p* = 0.004) and v) the development of moderate severe skin rash (*p*<0.0001). Finally, tumor differentiation (undifferentiated tumors) was significantly correlated with decreased median OS (Hazard Ratio: 1,9; *p* = 0.003).

In the multivariate analysis, *KRAS* (HR 4.3, *p*<0.0001), *BRAF* (HR 5.1, *p*<0.0001) mutation and low *EREG* mRNA expression (HR 1.6, *p* = 0.021) emerged as independent factors associated with reduced TTP. Furthermore, the absence of severe and moderate (grade 2–3) skin rash emerged as well, as an independent prognostic factor for decreased TTP (HR 4.0, *p*<0.0001) (**Table 5** **).** In addition, *KRAS* (HR 2.9, *p* = 0.01), *BRAF* (HR 3.0, *p* = 0.001) mutation and low *EREG* mRNA expression (HR 1.7, *p* = 0.021) emerged as independent factors associated with reduced OS. In addition, tumor differentiation grade 3 emerged, as well, as an independent prognostic factors for reduced OS (HR 2.2, *p* = 0.001). Furthermore, the absence of severe and moderate (grade 2–3) skin rash emerged as an independent prognostic factor for decreased OS (HR 3.7, *p*<0.0001, respectively) (**Table 5** **).**

**Table 5:** Multivariate analysis for Time to Tumor Progression and median Overall Survival.

**Progression-Free Survival**			
	**Hazard Ratio**	**95% CI** *	***p*** ** value**
***KRAS*** ** (mutant vs. WT** * **)**	**4.3**	**2.8–7.9**	**<0.0001**
***BRAF*** ** (mutant vs. WT** * **)**	**5.1**	**2.8–9.6**	**<0.0001**
***EREG*** ** mRNA expression (Low vs. High)**	**1.6**	**1.1–2.7**	**0.021**
**Exanthema (Grade 2–3 vs. 0–1)**	**4.0**	**2.52–6.4**	**<0.0001**
*PIK3CA (mutant vs. WT)*	1.9	0.9 – 3.7	0.115
*AREG* mRNA expression (Low vs. High)	1.4	0.9 – 1.9	0.149
PTEN expression (Loss vs. Functional)	1.3	0.6 – 1.6	0.252
**Overall Survival**			
***KRAS*** ** (mutant vs. WT** * **)**	**2.9**	**1.5–3.9**	**0.01**
***BRAF*** ** (mutant vs. WT** * **)**	**3.0**	**1.3–6.6**	**0.001**
***EREG*** ** mRNA expression (Low vs. High)**	**1.7**	**1.2–2.6**	**0.021**
**Tumor Grade (3 vs. 1–2)**	**2.2**	**1.4–3.5**	**0.001**
**Exanthema (Grade 2–3 vs. 0–1)**	**3.7**	**2.3–3.8**	**<0.0001**
*PIK3CA (mutant vs. WT)*	1.6	0.9–3.5	0.268
*AREG* mRNA expression (Low vs. High)	1.5	0.95–2.5)	0.123
PTEN expression (Loss vs. Functional)	1.5	0.8–2.6	0.192

*CI: Confidence Interval.

## Discussion

Following the discovery of *KRAS* mutations in association with anti-EGFR moAbs resistance, the *KRAS* mutational characterization of mCRC tumours is, currently, preformed in routine basis before any treatment decision. Although the presence of *KRAS* mutations is a specific predictive biomarker for lack of anti-EGFR moAbs efficacy [6]–[9], [14], [37] there is convincing evidence that additional genetic events are involved in this process, since approximately half of the *KRAS* wt patients are resistant to such a treatment [38]. In addition, several biomarkers have been proposed in association with *KRAS* mutations as predictive markers for the efficacy of the anti-EGFR moAbs including *BRAF* [19], [22] or *PIK3CA* mutations [21], EGFR ligands overexpression [23], [27], PTEN protein expression [28] and EGFR copy numbers [10], [11]. In the current study we evaluated the predictive significance of other common mutations observed in CRC in conjunction with PTEN protein expression and EGFR ligands (*EREG* and *AREG)* mRNA expression as well as the impact of skin rash in a cohort of patients with mCRC treated with anti-EGFR plus chemotherapy as salvage treatment. To the best of our knowledge this is the first study which combines all these parameters together. Patient's characteristics, the incidence of mutations and the treatment regimens were all typical for mCRC [22], [37]; therefore, the results of our analysis could serve as a useful guide for clinical practice.

The data presented here are consistent with previous reports demonstrating that *KRAS* and *BRAF* mutations are mutually exclusive; the prevalence of *BRAF* mutations (7.2%) is, practically, similar with that reported in other patients' series from a first-line setting [39], but higher than that described in heavily pre-treated colorectal cancer patients [21], [37], indicating that its prognostic significance mainly depends on the studied patients' population. The presence of *BRAF* mutations has been correlated with resistance to anti-EGFR moAbs treatment [19], [22], [34]. In accordance with these previous reports, in the current study we also observed that patients with tumours that harboured *BRAF* mutations had a significantly worse TTP and shorter OS compared to *BRAF* wt tumours. Furthermore, in our series of tumours, a statistically significant correlation was observed between *BRAF* mutations and the undifferentiated histological grade reflecting that this mutation seems to characterize a subgroup of patients with poor prognosis since they carry a significant higher risk of progression and death due to disease.

Mutations in *PIK3CA* and PTEN protein expression loss have also been suggested as biomarkers of anti-EGFR moAbs resistance. The role of *PIK3CA* mutational status on the anti-EGFR mutational status is conflicting. In the current study, *PIK3CA* mutations were identified in 11 tumours (9.8%) and, more especially, in exon 9 than in exon 20; this observation is in contrast with that observed in the Sartore-Bianchi's et al [21] cohort but in agreement with that reported by Prenen et al [26]. A significant negative correlation between *PIK3CA* mutations and response to anti-EGFR moAbs has been documented in the Sartore-Bianchi's et al [21] and the Perone's et al [30] reports, whereas, Prenen et al [26] could not find a clear association between the presence of *PIK3CA* mutation status and an impaired efficacy of anti-EGFR moAbs. Our data demonstrate that there was no significant correlation between the TTP and OS and the *PIK3CA* mutational status when the analysis was performed in the whole group of patients; however, when only *KRAS* wt patients were analyzed, *PIK3CA* mutational status was correlated with a significantly lower TTP. Nevertheless, this lower TTP could not be translated into differences in OS between wt *KRAS* patients with mutant and wt *PIK3CA* alleles in their primary tumours, as previously described by our group [22]. In a very recent study by De Roock et al [40], where a large cohort of patients has been evaluated, the role of *PIK3CA* mutational status has been more clearly revealed. Exon 9 and exon 20 *PIK3CA* mutations were able to be analyzed separately and, indeed, only exon 20 mutations were found to be associated with a worse outcome after cetuximab administration. This seems to be a possible explanation for the reported conflicting results published in the literature, since there could be more than one interpretation when two events (exon 9 and exon 20 mutations) have different and opposite effects. However, the lack of efficacy of EGFR moAbs which is observed in patients with mutant *KRAS* extends to other common mutations that deregulate the cellular signaling pathway, especially *BRAF* and, probably, *PIK3CA* [41].

The role of PTEN loss and consecutive over-activation of the AKT pathway and its evaluation is still under investigation, as far as response to anti-EGFR moAbs is concerned. Five relatively small, retrospective studies [26], [28]–[30] have provided evidence that PTEN status is associated with objective responses in cetuximab-treated mCRC patients suggesting that PTEN-positive tumours tend to have a better outcome than negative ones; however, another study failed to confirm this observation [21]. This probably could be due to several methodological differences such as the used anti-PTEN antibodies, the IHC scoring algorithms and cut-off criteria [31], [42]. In the present study, the significantly lower TTP which was observed in patients with wt *KRAS* and *PIK3CA* according to the down- and up-regulation of PTEN could not be translated into differences in OS. Nevertheless, since PTEN IHC is not yet adequately validated, it cannot be considered for immediate routine clinical use, but, it should be kept in mind in the planning process of prospective biomarkers studies.

EGFR ligands *AREG* and *EREG* were quite recently found by biomarker exploratory analysis using Affimetrix to be the top genes associated with efficacy to anti-EGFR moAbs [27]. In the group of patients with wt *KRAS* we found a statistically significant correlation of *AREG* and especially *EREG* mRNA overexpression with increased TTP and OS in accordance with previous reports [23]. Our data also seem to identify a subgroup of *KRAS* wt patients who could be considered to more EGFR-dependent and, thus, have a higher probability of responding to EGFR inhibition as already previously has been reported [23]. Patients whose tumours were characterized by ligands' downregulation behaved like *KRAS* mutants upon treatment with anti-EGFR moAbs.

The most frequently reported side effect of EGFR inhibitors is a dose-dependent acneiform skin rash occurring in more than 50% of patients [42]. A number of studies have suggested that from a clinical point of view, the severity of skin rash is positively correlated with clinical outcome (response rates, progression free survival and OS) and, thus, it could be used in order to distinguish mCRC patients more likely to be sensitive to anti-EGFR treatment [2], [32], [42]. Particularly, the analysis of the PRIME trial showed that the patients with *KRAS* mutated tumours and moderate or severe skin rash presented better outcome in comparison with those with *KRAS* wt tumours and no or mild skin rash [32]. In our study as well, mCRC patients with severe and moderate skin rash presented significantly higher TTP and OS compared with those with mild and no rash. Indeed, in the multivariate analysis the absence of severe and moderate (grade 3 and 2) skin rash formation emerged as an independent predictive factor for reduced TTP and OS. Although skin toxicity seems to be an important clinical surrogate marker of anti-EGFR moAbs efficacy, the biological correlation is still unknown and the elucidation of the biologic mechanisms will be of great value.

The multivariate analysis revealed that the presence of *KRAS* or *BRAF* mutations and *EREG* downregulation are the only biomarkers which are independent prognostic factors for decreased TTP and OS. In a recently published study, the mutational analysis of *KRAS, BRAF, NRAS* and *PIK3CA* exon 20, in that specific order, has been proposed as the most effective approach [40]. The common finding between the two studies is that multigene models seem to be more effective than single-gene analysis for the selection of patients who could gain the maximum benefit from the administration of anti-EGFR moAbs. The important issue of cost for the molecular analysis and the limited amount of tumour cells available in FFPE specimens for all potential biomarkers testing could be tackled with the development of multiplex assays [43]. Furthermore, the severity of skin rash during the treatment with anti-EGFR mo-Abs has been constantly reported as a predictive factor for response and survival [2], [16], and this was also the case in the present study, since the severity of skin rash was an independent predictive factor for TTP and OS. The biologic mechanism which links the development of severe skin rash and tumor response is not yet elucidated, and very few data are published regarding this issue [44].

In summary, the genetics underpinnings of CRC are established [45] and the results of the present study support the idea that advanced application of CRC genetic profiling could lead to informed treatment decisions. Despite the fact that the results of a retrospective study should be interpreted with caution, it seems that the determination of the *KRAS*-*BRAF* mutational status, with additional screening of CRC tumours for their *EREG* mRNA expression, could help stratify patients likely to benefit from a regimen containing an anti-EGFR moAb. Studies which focus in the elucidation of the mechanism which links the development of skin rash with tumors response are urgently warranted. Nevertheless, since most available data come from retrospective studies, validation in prospective randomized clinical trials is imperative in order to formally confirm the predictive and prognostic value of these biomarkers.

## References

[1] Jemal A, Siegel R, Ward E, Hao Y, Xu J (2009). Cancer statistics, 2009.. CA Cancer J Clin.

[2] Cunningham D, Humblet Y, Siena S, Khayat D, Bleiberg H (2004). Cetuximab monotherapy and cetuximab plus irinotecan in irinotecan-refractory metastatic colorectal cancer.. N Engl J Med.

[3] Saltz LB, Cox JV, Blanke C, Rosen LS, Fehrenbacher L (2000). Irinotecan plus fluorouracil and leucovorin for metastatic colorectal cancer. Irinotecan Study Group.. N Engl J Med.

[4] Saltz LB, Meropol NJ, Loehrer PJ, Needle MN, Kopit J (2004). Phase II trial of cetuximab in patients with refractory colorectal cancer that expresses the epidermal growth factor receptor.. J Clin Oncol.

[5] Van CE, Peeters M, Siena S, Humblet Y, Hendlisz A (2007). Open-label phase III trial of panitumumab plus best supportive care compared with best supportive care alone in patients with chemotherapy-refractory metastatic colorectal cancer.. J Clin Oncol.

[6] Amado RG, Wolf M, Peeters M, Van CE, Siena S (2008). Wild-type KRAS is required for panitumumab efficacy in patients with metastatic colorectal cancer.. J Clin Oncol.

[7] De RW, Piessevaux H, De SJ, Janssens M, De HG (2008). KRAS wild-type state predicts survival and is associated to early radiological response in metastatic colorectal cancer treated with cetuximab.. Ann Oncol.

[8] Lievre A, Bachet JB, Le CD, Boige V, Landi B (2006). KRAS mutation status is predictive of response to cetuximab therapy in colorectal cancer.. Cancer Res.

[9] Lievre A, Bachet JB, Boige V, Cayre A, Le CD (2008). KRAS mutations as an independent prognostic factor in patients with advanced colorectal cancer treated with cetuximab.. J Clin Oncol.

[10] Moroni M, Veronese S, Benvenuti S, Marrapese G, Sartore-Bianchi A (2005). Gene copy number for epidermal growth factor receptor (EGFR) and clinical response to antiEGFR treatment in colorectal cancer: a cohort study.. Lancet Oncol.

[11] Sartore-Bianchi A, Moroni M, Veronese S, Carnaghi C, Bajetta E (2007). Epidermal growth factor receptor gene copy number and clinical outcome of metastatic colorectal cancer treated with panitumumab.. J Clin Oncol.

[12] Bokemeyer C, Bondarenko I, Makhson A, Hartmann JT, Aparicio J (2009). Fluorouracil, leucovorin, and oxaliplatin with and without cetuximab in the first-line treatment of metastatic colorectal cancer.. J Clin Oncol.

[13] Douillard J, Siena S, Cassidy J, Tabernero J, Burkes R (2009). Randomized phase 3 study of panitumumab with FOLFOX4 compared to FOLFOX4 alone as 1st-line treatment (tx) for metastatic colorectal cancer (mCRC): the PRIME trial.. AnnOncol supp.

[14] Karapetis CS, Khambata-Ford S, Jonker DJ, O'Callaghan CJ, Tu D (2008). K-ras mutations and benefit from cetuximab in advanced colorectal cancer.. N Engl J Med.

[15] Tol J, Koopman M, Cats A, Rodenburg CJ, Creemers GJ (2009). Chemotherapy, bevacizumab, and cetuximab in metastatic colorectal cancer.. N Engl J Med.

[16] Van CE, Kohne CH, Hitre E, Zaluski J, Chang Chien CR (2009). Cetuximab and chemotherapy as initial treatment for metastatic colorectal cancer.. N Engl J Med.

[17] Benvenuti S, Sartore-Bianchi A, Di NF, Zanon C, Moroni M (2007). Oncogenic activation of the RAS/RAF signaling pathway impairs the response of metastatic colorectal cancers to anti-epidermal growth factor receptor antibody therapies.. Cancer Res.

[18] Di FF, Blanchard F, Charbonnier F, Le PF, Lamy A (2007). Clinical relevance of KRAS mutation detection in metastatic colorectal cancer treated by Cetuximab plus chemotherapy.. Br J Cancer.

[19] Di NF, Martini M, Molinari F, Sartore-Bianchi A, Arena S (2008). Wild-type BRAF is required for response to panitumumab or cetuximab in metastatic colorectal cancer.. J Clin Oncol.

[20] Laurent-Puig P, Cayre A, Manceau G, Buc E, Bachet JB (2009). Analysis of PTEN, BRAF, and EGFR status in determining benefit from cetuximab therapy in wild-type KRAS metastatic colon cancer.. J Clin Oncol.

[21] Sartore-Bianchi A, Martini M, Molinari F, Veronese S, Nichelatti M (2009). PIK3CA mutations in colorectal cancer are associated with clinical resistance to EGFR-targeted monoclonal antibodies.. Cancer Res.

[22] Souglakos J, Philips J, Wang R, Marwah S, Silver M (2009). Prognostic and predictive value of common mutations for treatment response and survival in patients with metastatic colorectal cancer.. Br J Cancer.

[23] Jacobs B, De RW, Piessevaux H, Van OR, Biesmans B (2009). Amphiregulin and epiregulin mRNA expression in primary tumors predicts outcome in metastatic colorectal cancer treated with cetuximab.. J Clin Oncol.

[24] Jhawer M, Goel S, Wilson AJ, Montagna C, Ling YH (2008). PIK3CA mutation/PTEN expression status predicts response of colon cancer cells to the epidermal growth factor receptor inhibitor cetuximab.. Cancer Res.

[25] Ogino S, Nosho K, Kirkner GJ, Shima K, Irahara N (2009). PIK3CA mutation is associated with poor prognosis among patients with curatively resected colon cancer.. J Clin Oncol.

[26] Prenen H, De SJ, Jacobs B, De RW, Biesmans B (2009). PIK3CA mutations are not a major determinant of resistance to the epidermal growth factor receptor inhibitor cetuximab in metastatic colorectal cancer.. Clin Cancer Res.

[27] Khambata-Ford S, Garrett CR, Meropol NJ, Basik M, Harbison CT (2007). Expression of epiregulin and amphiregulin and K-ras mutation status predict disease control in metastatic colorectal cancer patients treated with cetuximab.. J Clin Oncol.

[28] Loupakis F, Pollina L, Stasi I, Ruzzo A, Scartozzi M (2009). PTEN expression and KRAS mutations on primary tumors and metastases in the prediction of benefit from cetuximab plus irinotecan for patients with metastatic colorectal cancer.. J Clin Oncol.

[29] Frattini M, Saletti P, Romagnani E, Martin V, Molinari F (2007). PTEN loss of expression predicts cetuximab efficacy in metastatic colorectal cancer patients.. Br J Cancer.

[30] Perrone F, Lampis A, Orsenigo M, Di BM, Gevorgyan A (2009). PI3KCA/PTEN deregulation contributes to impaired responses to cetuximab in metastatic colorectal cancer patients.. Ann Oncol.

[31] Prenen H, Tejpar S, Van CE (2009). Impact of molecular markers on treatment selection in advanced colorectal cancer.. Eur J Cancer.

[32] Douillard J, Cassidy J, Jassem J, Rivera F, Kocakova I (2010). Randomized, open label, phase III study of panitumumab (pmab) with FOLFOX4 versus FOLFOX4 alone as first-line treatment (tx) for metastatic colorectal cancer (mCRC): Efficacy by skin toxicity (ST).. J Clin Oncol.

[33] Benlloch S, Paya A, Alenda C, Bessa X, Andreu M (2006). Detection of BRAF V600E mutation in colorectal cancer: comparison of automatic sequencing and real-time chemistry methodology.. J Mol Diagn.

[34] Saridaki Z, Papadatos-Pastos D, Tzardi M, Mavroudis D, Bairaktari E (2010). BRAF mutations, microsatellite instability status and cyclin D1 expression predict metastatic colorectal patients' outcome.. Br J Cancer.

[35] Papadaki C, Mavroudis D, Trypaki M, Koutsopoulos A, Stathopoulos E (2009). Tumoral expression of TXR1 and TSP1 predicts overall survival of patients with lung adenocarcinoma treated with first-line docetaxel-gemcitabine regimen.. Clin Cancer Res.

[36] Torres J, Navarro S, Rogla I, Ripoll F, Lluch A (2001). Heterogeneous lack of expression of the tumour suppressor PTEN protein in human neoplastic tissues.. Eur J Cancer.

[37] Richman SD, Seymour MT, Chambers P, Elliott F, Daly CL (2009). KRAS and BRAF mutations in advanced colorectal cancer are associated with poor prognosis but do not preclude benefit from oxaliplatin or irinotecan: results from the MRC FOCUS trial.. J Clin Oncol.

[38] Wong R, Cunningham D (2008). Using predictive biomarkers to select patients with advanced colorectal cancer for treatment with epidermal growth factor receptor antibodies.. J Clin Oncol.

[39] Saridaki Z, Georgoulias V, Souglakos J (2010). Mechanisms of resistance to anti-EGFR monoclonal antibody treatment in metastatic colorectal cancer.. World J Gastroenterol.

[40] De RW, Claes B, Bernasconi D, De SJ, Biesmans B (2010). Effects of KRAS, BRAF, NRAS, and PIK3CA mutations on the efficacy of cetuximab plus chemotherapy in chemotherapy-refractory metastatic colorectal cancer: a retrospective consortium analysis.. Lancet Oncol.

[41] Razis E, Briasoulis E, Vrettou E, Skarlos DV, Papamichael D (2008). Potential value of PTEN in predicting cetuximab response in colorectal cancer: an exploratory study.. BMC Cancer.

[42] Segaert S, Chiritescu G, Lemmens L, Dumon K, Van CE (2009). Skin toxicities of targeted therapies.. Eur J Cancer.

[43] Lurkin I, Stoehr R, Hurst CD, van Tilborg AAG, Knowles MA (2010). Two multiplex assays that simultaneously identify 22 possible mutation sites in KRAS, BRAF, NRAS and PIK3CA genes.. PLoSone.

[44] Tabernero J, Cervantes A, Rivera F, Martinelli E, Rojo F (2010). Pharmacogenomic and pharmacoproteomic studies of cetuximab in metastatic colorectal cancer: biomarker analysis of a phase I dose-escalation study.. J Clin Oncol.

[45] Wood LD, Parsons DW, Jones S, Lin J, Sjoblom T (2007). The genomic landscapes of human breast and colorectal cancers.. Science.

